# Volume Vascular Pulmonar Estimado por *Software* Automatizado é um Preditor de Mortalidade após Embolia Pulmonar Aguda

**DOI:** 10.36660/abc.20190392

**Published:** 2020-11-01

**Authors:** Leonardo Soriano, Marcel Koenigkam Santos, Danilo Tadeu Wada, Kelvin Vilalva, Talita Tavares Castro, Oliver Weinheimer, Valdair Francisco Muglia, Antonio Pazin, Carlos Henrique Miranda

**Affiliations:** 1 Universidade de São Paulo Faculdade de Medicina de Ribeirão Preto Departamento de Clínica Médica Ribeirão PretoSP Brasil Universidade de São Paulo Faculdade de Medicina de Ribeirão Preto - Divisão de Medicina de Emergência do Departamento de Clínica Médica, Ribeirão Preto, SP - Brasil; 2 Universidade de São Paulo Faculdade de Medicina de Ribeirão Preto Departamento de Imagens Médicas Ribeirão PretoSP Brasil Universidade de São Paulo Faculdade de Medicina de Ribeirão Preto - Divisão de Radiologia do Departamento de Imagens Médicas, Hematologia e Oncologia Clínica, Ribeirão Preto, SP - Brasil; 3 University Hospital Heidelberg Translational Lung Research Centre Heidelberg Department of Diagnostic and Interventional Radiology Heidelberg Alemanha University Hospital Heidelberg - Department of Diagnostic and Interventional Radiology and Translational Lung Research Centre Heidelberg (TLRC) - German Lung Research Centre (DZL), Heidelberg – Alemanha

**Keywords:** Embolia Pulmonar, Tomografia Computadorizada, Prognóstico, Diagnóstico por Imagem, Circulação Pulmonar, Serviços Médicos de Emergência, Mortalidade

## Abstract

**Fundamento:**

A embolia pulmonar aguda (EPA) tem desfecho clínico variável. A angiotomografia computadorizada (angio-CT) é considerada o padrão-ouro para o diagnóstico.

**Objetivo:**

Avaliar se o volume vascular pulmonar (VVP) quantificado por *software* automatizado é um preditor de mortalidade após EPA.

**Métodos:**

Estudo de coorte retrospectivo no qual a imagem da angio-CT de 61 pacientes com EPA foi reanalisada. O VVP e o volume pulmonar (VP) foram estimados automaticamente pelo *software* Yacta. Calculamos o VVP ajustado pela razão: VVP(cm^3^)/VP(litros). Parâmetros prognósticos clássicos da angio-CT (carga embólica; razão do diâmetro do ventrículo direito/ventrículo esquerdo; razão do diâmetro da artéria pulmonar/aorta; desvio do septo interventricular; infarto pulmonar e refluxo de contraste na veia hepática) foram avaliados. A mortalidade em 1 mês foi o desfecho analisado. Consideramos um valor de p <0,05 como estatisticamente significativo.

**Resultados:**

Sete mortes (11%) ocorreram entre os 61 pacientes durante 1 mês de seguimento. O VVP ajustado <23cm^3^/L foi um preditor independente de mortalidade na análise univariada (*odds ratio* [OR]: 26; intervalo de confiança de 95% [IC95%]: 3-244; p=0,004) e na análise multivariada (OR ajustado: 19 [IC95%: 1,3-270]; p=0,03). Os parâmetros clássicos da angio-CT não foram associados à mortalidade em 1 mês nesta amostra. O VVP ajustado <23cm^3^/L apresentou sensibilidade de 86%, especificidade de 82%, valor preditivo negativo de 94% e valor preditivo positivo de 64% para identificação dos pacientes que morreram.

**Conclusão:**

VVP ajustado <23cm^3^/L foi um preditor independente de mortalidade após EPA. Esse parâmetro mostrou melhor desempenho prognóstico do que os outros achados clássicos da angio-CT. (Arq Bras Cardiol. 2020; 115(5):809-818)

## Introdução

A embolia pulmonar aguda (EPA) é uma causa importante de dispneia e dor torácica no departamento de emergência.^1^ O prognóstico após um evento deste é extremamente variável. A maioria dos pacientes tem um excelente desfecho clínico; no entanto, alguns podem ter um desfecho clínico catastrófico, evoluindo para choque circulatório, parada cardíaca e morte.^2^ Devido a essa apresentação clínica heterogênea, alguns parâmetros são utilizados para a estratificação prognóstica, a fim de permitir uma vigilância mais intensiva entre os pacientes com maior probabilidade de complicações. Atualmente, a angiotomografia computadorizada (angio-CT) é considerada o método padrão-ouro para o diagnóstico. Por esse motivo, os parâmetros desse exame de imagem são avaliados para auxiliar na estratificação prognóstica e na tomada de decisão sobre o tratamento.^3^^–^^5^

O parâmetro da angio-CT mais frequentemente utilizado para a estratificação prognóstica é a dilatação do ventrículo direito, que pode ser identificada por meio da razão do diâmetro do ventrículo direito (VD)/ventrículo esquerdo (VE) ≥1,^6^ ou da carga embólica, quantificada manualmente conforme descrito por Qanadli, maior que 40%.^7^ No entanto, na prática clínica, esses parâmetros isolados apresentam fraca associação com mortalidade e desenvolvimento de choque circulatório. Por esse motivo, as diretrizes recomendam que esses parâmetros sejam utilizados apenas combinados com outros marcadores prognósticos, como, por exemplo, a dosagem de troponina e da porção N-terminal do peptídio natriurético do tipo B (NT-proBNP).^8^

O objetivo dessa investigação foi avaliar se a quantificação automática do volume vascular pulmonar (VVP) por meio da angio-CT é um preditor de mortalidade após EPA e comparar o seu desempenho prognóstico com outros parâmetros clássicos obtidos a partir desse método de imagem.

## Métodos

Estudo de coorte retrospectivo de centro único que incluiu pacientes com diagnóstico primário de EPA admitidos em nosso departamento de emergência. Nosso hospital é voltado exclusivamente para emergências de alta complexidade, com cerca de 3.000 consultas médicas por mês. Este estudo foi aprovado pelo Comitê de Ética em Pesquisa de nossa instituição e seguiu a Declaração de Helsinque.

### Pacientes

Os prontuários de pacientes adultos (> 18 anos) admitidos de janeiro de 2009 a dezembro de 2015 com o diagnóstico primário de EPA registrado na alta hospitalar pelos códigos I26.0 (embolia pulmonar com *cor pulmonale* agudo) e I26.9 (embolia pulmonar sem *cor pulmonale* agudo) da Classificação Estatística Internacional de Doenças (CID-10) foram revisados. O diagnóstico definitivo de EPA foi definido como a presença de condição clínica compatível associada a pelo menos um critério, que poderia ser: angio-CT com defeitos de enchimento intraluminal; cintilografia de ventilação e perfusão pulmonar com defeitos de perfusão em áreas ventiladas (alta probabilidade); angiografia pulmonar convencional com defeito de enchimento intraluminal; ultrassonografia de membro inferior compatível com trombose venosa profunda; ou necropsia com alta carga embólica na artéria pulmonar sem evidência de outro diagnóstico alternativo.

Dados demográficos e clínicos foram obtidos por meio da revisão de prontuários. Utilizamos os diagnósticos referidos pelo paciente e registrados no prontuário. O desfecho avaliado nesta investigação foi a mortalidade por todas as causas em um mês. Para os pacientes que receberam alta antes de completar 30 dias, um enfermeiro da unidade de pesquisa clínica de nossa instituição, treinado para avaliar a sobrevida, fez uma ligação telefônica, e, quando verificada a ocorrência de óbito, solicitou-se a indicação da data do evento.

### Técnica e interpretação da angio-CT

A angio-CT foi realizada em *scanners* de tomografia computadorizada com múltiplos detectores (TCMD), e as imagens volumétricas foram obtidas após administração intravenosa de contraste iodado usando injeção de *bolus* único seguido de solução salina e técnica de detecção de *bolus* para identificar a contrastação da artéria pulmonar. Outros parâmetros típicos utilizados foram: espessura do corte ≤2,5 mm, intervalo de reconstrução de 1 mm, kVp de 120, referência mAs de 150-220, rotação do pórtico de 0,3 a 0,7 s. As aquisições volumétricas foram reconstruídas com filtro macio e duro. Dois radiologistas de tórax analisaram novamente as imagens após recuperá-las no formato DICOM (*digital imaging and communication in medicine*), usando estações de trabalho calibradas e dedicadas. Ambos os radiologistas eram cegos para a evolução clínica desses pacientes.

Analisamos os parâmetros prognósticos clássicos da angio-CT descritos na literatura médica. A razão do diâmetro axial do VD/VE foi obtida medindo os eixos curtos dos VD e VE no plano axial no terço posterior. Foi utilizado como ponto de corte da relação do diâmetro VD/VE o valor de 1, conforme recomendado na literatura. Tanto o diâmetro transversal da artéria pulmonar (AP) principal quanto o da aorta ascendente ao mesmo nível foram medidos. Considerou-se abaulamento do septo interventricular se houvesse achatamento septal e desvio de septo convexo em direção ao ventrículo esquerdo. A presença de refluxo de contraste nas veias hepáticas também foi avaliada. A presença de infarto pulmonar foi definida por uma opacidade parenquimatosa subpleural com bordas convexas e abauladas e ápice direcionado para o hilo. A carga embólica foi calculada pelo método descrito por Qanadli et al^.^^7^ Considerou-se um índice superior a 60% como indicativo de uma alta carga embólica.

A análise vascular quantitativa da imagem foi realizada com o programa acadêmico Yacta (Universidade de Heidelberg, Heidelberg, Alemanha) versão 2.7. O *software* Yacta funciona de forma totalmente automática, sem necessidade de intervenção do usuário em nenhum estágio do processo. A análise da imagem dura cerca de 10 minutos. Inicialmente, esse *software* separa anatomicamente as vias aéreas, os vasos sanguíneos, os pulmões e seus lobos, fornecendo volumes e densidades pulmonares, juntamente com o volume dos vasos sanguíneos. Tal *software* usa um coeficiente de atenuação de –500 HU como o limite padrão para detecção de vasos. Nos pulmões com coeficientes de atenuação modificados, o Yacta calcula um novo limiar com base no histograma. Os *voxels* intrapulmonares com coeficientes acima do limiar calculado são então marcados e considerados como vasos se os mesmos apresentarem uma extensão tridimensional maior que 100 mm^3^.^9^^,^^10^ O software Yacta estimou o volume pulmonar (VP) em litros (L) e o VVP em cm^3^. Como o VVP apresenta variação de acordo com o tamanho do pulmão, realizamos um ajuste por meio da razão: VVP (cm^3^)/VP (L).

### Análise estatística

Utilizamos o teste de Shapiro-Wilk para avaliar o tipo de distribuição das variáveis. As variáveis categóricas foram expressas como porcentagem. As variáveis contínuas com distribuição normal foram expressas como média e desvio padrão, e as demais variáveis foram expressas como mediana e intervalo interquartil (percentil 25, percentil 75). Utilizamos o teste do qui-quadrado para comparar duas variáveis categóricas; o teste t de Student não pareado para comparar duas variáveis contínuas com distribuição normal; e o teste de Mann-Whitney para comparar duas variáveis contínuas com distribuição não normal. Na análise univariada, calculou-se o *odds ratio* (OR) e seu respectivo intervalo de confiança de 95% (IC95%) para cada parâmetro, seguido pelo teste do qui-quadrado. Para análise multivariada, foi utilizado um modelo de regressão logística com ajuste para as variáveis: idade, *pulmonar embolism severety index* (PESI) escore, frequência respiratória, parada cardíaca e choque circulatório. Utilizamos o teste de Spearman para avaliar a correlação entre duas variáveis contínuas. A área sob a curva (ASC) ROC (*Operating Characteristic Curve*) foi usada para comparar a acurácia prognóstica de cada parâmetro da angio-CT. Utilizamos o índice de Youden para determinar o melhor ponto de corte do VVP ajustado para identificar os pacientes que morreram. Para os demais parâmetros da angio-CT, utilizamos o ponto de corte padronizado na literatura médica. Na análise de sobrevida, comparamos as curvas de Kaplan-Meier por meio do teste de log-rank. Um valor de p <0,05 foi considerado estatisticamente significativo. O *software* STATA 13.1 (College Station, TX, EUA) foi utilizado para análise estatística.

## Resultados

**Figura 1:**
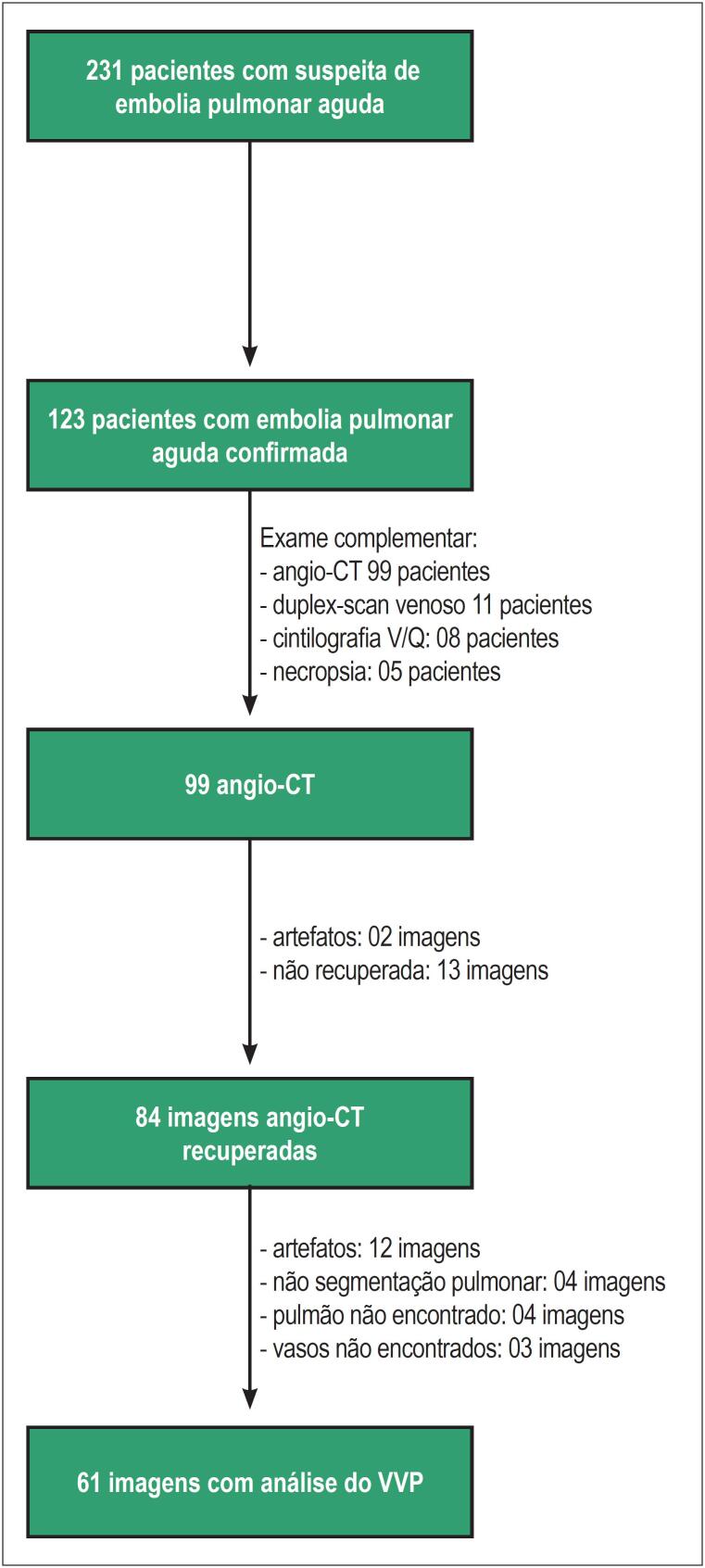
Fluxograma mostrando os critérios de seleção dos pacientes incluídos nesta investigação. V/Q: ventilação/perfusão; angio-CT: angiotomografia computadorizada; VVP: volume vascular pulmonar.

Entre 231 indivíduos com suspeita de EPA avaliados no departamento de emergência, esse diagnóstico foi confirmado em 123 pacientes (53%). O diagnóstico foi confirmado por meio da angio-CT em 99 pacientes (80%). Destes pacientes submetidos à angio-CT, a imagem foi recuperada para reanálise em 84 deles. A determinação automatizada do VVP pelo *software* Yacta foi possível em 61 destas imagens recuperadas. O fluxograma dos pacientes incluídos nessa investigação e os motivos que impossibilitaram a análise pelo *software* Yacta são apresentados na Figura 1.

As características desses 61 pacientes são mostradas na Tabela 1. Entre esses pacientes, 7 (11%) morreram durante o seguimento de 1 mês. Na comparação entre não sobreviventes (n=7) e sobreviventes (n=54), não foram observadas diferenças significativas entre os dois grupos, exceto, uma frequência respiratória mais elevada no grupo de não sobreviventes (31 ± 7 ciclos/min *vs.* 33 ± 7 ciclos/min, p=0,01).

**Tabela 1:** Características dos pacientes incluídos nesta investigação divididos de acordo com a sobrevida em 1 mês

Características	Sobreviventes n=54	Não sobreviventes n=7	p
**Demográficas**			
Idade, anos (média ± dp)	54±16	61±17	0,34
Idade >65 anos, n. (%)	19(35)	3(43)	0,69
Gênero masculino, n. (%)	24(44)	2(29)	0,42
**Apresentação clínica**			
Parada cardíaca, n. (%)	2(04)	1(14)	0,22
Choque circulatório, n. (%)	5(09)	2(28)	0,13
Dispneia, n. (%)	46(85)	7(100)	0,27
Hemoptise, n. (%)	7(13)	0(00)	0,31
Síncope, n. (%)	13(24)	0(00)	0,14
Tosse, n. (%)	20(37)	3(43)	0,76
Dor torácica pleurítica, n. (%)	17(31)	4(57)	0,18
Febre, n. (%)	7(13)	2(28)	0,27
Escore de Wells (mediana, IQ)	4,5 (3,0-7,0)	4,0 (1,5-4,5)	0,17
Escore PESI (mediana, IQ)	78 (65-108)	97 (95-108)	0,13
Duração dos sintomas, dias (mediana, IQ)	3(1-6)	2(1-6)	0,29
**Fatores predisponentes**			
Trombose venosa prévia, n. (%)	11(20)	0(00)	0,19
Câncer ativo, n. (%)	4(07)	2(28)	0,07
Cirurgia recente, n. (%)	7(13)	0(00)	0,31
Imobilização, n. (%)	13(24)	1(14)	0,56
Fratura óssea, n. (%)	7(13)	0(00)	0,31
AVE prévio, n. (%)	7(13)	1(14)	0,92
Anticoncepcional oral, n. (%)	7(13)	0(00)	0,31
Obesidade, n. (%)	23(43)	3(43)	0,91
Insuficiência cardíaca, n. (%)	7(13)	0(00)	0,31
DPOC, n. (%)	4(07)	1(14)	0,53
Trombofilia, n. (%)	5(09)	1(14)	0,67
**Exame físico**			
Frequência cardíaca; bpm, (média±dp)	94±16	106±23	0,07
Frequência respiratória, ciclos/min (média ± dp)	23±7	31±7	0,01
Frequência respiratória >20 ciclos/min, n. (%)	36(67)	6(86)	0,12
PAS, mmHg (média ± dp)	123±28	113±14	0,37
PAD, mmHg (média ± dp)	75±14	69±19	0,32
**Exames laboratoriais**			
Creatinina, mg/dL (média ± dp)	1,08±0,27	1,16±0,83	0,59
Hemoglobina, g/dL (média ± dp)	13±2	12±3	0,05
Saturação arterial oxigênio, % (média ± dp)	92±7	87±8	0,09
Troponina I, μg/L (média ± dp)	0,16±0,29	0,13±0,12	0,79
NT-proBNP, μg/L (média ± dp)	2604±3040	3433±2343	0,60
**Tratamento**			
Trombolítico, n. (%)	14(26)	2(29)	0,88
Heparina não fracionada, n. (%)	7(13)	1(14)	0,81
Heparina de baixo peso molecular, n. (%)	38(70)	6(86)	0,12

dp: desvio-padrão; IQ: intervalo interquartil; PESI: pulmonary embolism severity index; AVE: acidente vascular encefálico; DPOC: doença pulmonar obstrutiva crônica; PAS: pressão arterial sistólica; PAD: pressão arterial diastólica; NT-proBNP: N-terminal peptídio natriurético do tipo B.

Na análise dos parâmetros da angio-CT, o VVP e o VVP ajustado foram significativamente menores no grupo de não sobreviventes em comparação ao grupo de sobreviventes (56 ± 24 cm^3^ *vs.* 88 ± 32 cm^3^, p=0,015 e 21 ± 6 cm^3^/L *vs.* 30 ± 7 cm^3^/L, p=0,001, respectivamente). Os demais parâmetros avaliados pela angio-CT (carga embólica, razão do diâmetro axial VD/VE, razão do diâmetro AP/Aorta, abaulamento do septo interventricular, infarto pulmonar e refluxo de contraste na veia hepática) não diferiram significativamente entre os dois grupos (Tabela 2).

**Tabela 2:** Achados da angiotomografia computadorizada (angio-CT) de acordo com a sobrevida em 1 mês

Parâmetro	Sobreviventes n=54	Não sobreviventes n=7	p
**Parâmetros do Yacta**			
Volume pulmonar (L), média ± dp	2,91±0,90	2,73±1,31	0,64
Volume vascular pulmonar (cm^3^), média ± dp	88±32	56±24	0,01
Volume vascular pulmonar ajustado (cm^3^/L), média ± dp	30±7	21±6	0,001
**Parâmetros clássicos**			
Carga embólica (%), média ± dp	47±21	40±26	0,40
Êmbolo central, n. (%)	5 (09)	2(28)	0,13
Êmbolo bilateral, n. (%)	45(83)	5(72)	0,59
Êmbolo unilateral, n. (%)	4(08)	0(00)	1,00
Diâmetro axial VD/VE, média ± dp	1,20±0,36	1,25±0,28	0,74
Diâmetro axial VD/VE >1, n. (%)	36(67)	6(86)	0,30
Diâmetro AP/Aorta, média ± dp	0,91±0,17	0,91±0,90	0,92
Desvio do septo interventricular, n. (%)	32(59)	5(71)	0,53
Infarto pulmonar, n. (%)	25(46)	2(29)	0,37
Refluxo de contraste para veia hepática, n. (%)	20(37)	3(43)	0,76

dp: desvio-padrão; VD: ventrículo direito; VE: ventrículo esquerdo; AP: artéria pulmonar.

**Figura 2:**
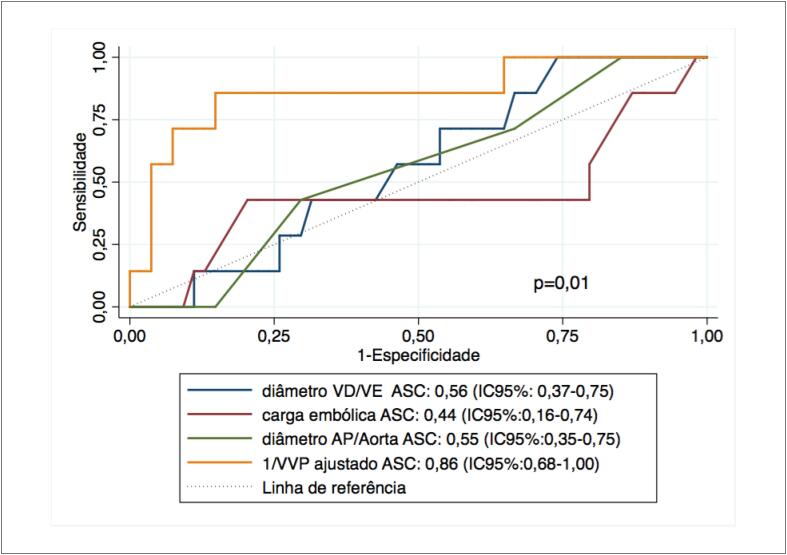
Curvas ROC mostrando o desempenho prognóstico dos parâmetros contínuos da angiotomografia computadorizada (carga embólica, razão do diâmetro VD/VE, razão do diâmetro AP/aorta) em comparação com o volume vascular pulmonar (VVP) ajustado na predição de mortalidade em 1 mês após embolia pulmonar aguda. VD: ventrículo direito; VE: ventrículo esquerdo; ASC: área sob a curva; AP: artéria pulmonar.

Na análise através da área sob a curva ROC (ASC), o 1/VVP ajustado mostrou a melhor acurácia prognóstica, com uma ASC de 0,86 (IC95%: 0,68-1,00) em comparação com as outras variáveis contínuas (razão do diâmetro VD/VE com ASC de 0,56 [IC95%: 0,37-0,75], diâmetro AP/Aorta com ASC de 0,55 [IC95%: 0,35-0,75] e carga embólica com ASC de 0,44 [IC95%: 0,16-0,74]), p <0,01 (Figura 2).

O melhor ponto de corte do VVP ajustado para determinar a mortalidade em um mês foi 23 cm^3^/L (sensibilidade: 86% [IC95%: 42-99], especificidade: 82% [IC95%: 69-91], valor preditivo positivo: 64% [IC95%: 49-77] e valor preditivo negativo: 94% [IC95%: 70-99]).

Na análise univariada, o VVP ajustado <23 cm^3^/L (*odds ratio* [OR]: 26 [IC95%: 3-244], p=0,004) e a frequência respiratória (OR: 1,1 [IC95%: 1,01- 1,26], p=0,03) foram preditores de mortalidade em 1 mês. Na análise multivariada, apenas o VVP ajustado <23 cm^3^/L permaneceu como preditor independente de mortalidade em um mês (OR ajustado: 19 [IC95%: 1,3-270], p=0,03). Os parâmetros prognósticos clássicos da angio-CT não foram associados à mortalidade em 1 mês nesta amostra (Tabela 3).

**Tabela 3:** Preditores de mortalidade em 1 mês após embolia pulmonar aguda na análise univariada e multivariada

Parâmetros	Univariada				Multivariada		
	OR	IC 95%	p		OR	IC 95%	p
**Demográficos/Clínicos**							
Idade	1,0	0,97- 1,0	0,34				
Gênero	0,5	0,09-2,8	0,43				
Câncer ativo	5,0	0,73-34,5	0,10				
Choque circulatório	3,9	0,60-25,7	0,15				
Parada cardíaca	4,3	0,34-55,2	0,26				
Frequência cardíaca	1,0	0,99-1,1	0,09				
Frequência respiratória	1,1	1,01-1,26	0,03		1,56	0,95-2,57	0,08
Escore PESI	1,0	0,99-1,00	0,12				
**Angio-CT**							
Volume vascular pulmonar ajustado ≤ 23 cm^3^/L	26,0	3,0-244	0,004		19,0	1,3-279,0	0,03
Carga embólica	0,9	0,95-1,0	0,44				
Carga embólica ≥ 40%	0,5	0,0-2,3	0,36				
Carga embólica ≥ 60%	2,6	0,5-13,4	0,24				
Diâmetro VD/VE	1,5	0,2-12,6	0,73				
Diâmetro VD/VE ≥1	3,0	0,3-26,8	0,32				
Desvio do septo interventricular	1,7	0,3-9,6	0,53				
Infarto pulmonar	0,5	0,8-2,6	0,38				
Refluxo de contraste para veia hepática	1,3	0,2-6,3	0,76				

Angio-CT: angiotomografia computadorizada; OR: odds ratio; IC: intervalo de confiança; PESI: pulmonary embolism severity index; VD: ventrículo direito; VE: ventrículo esquerdo.

**Figura 3:**
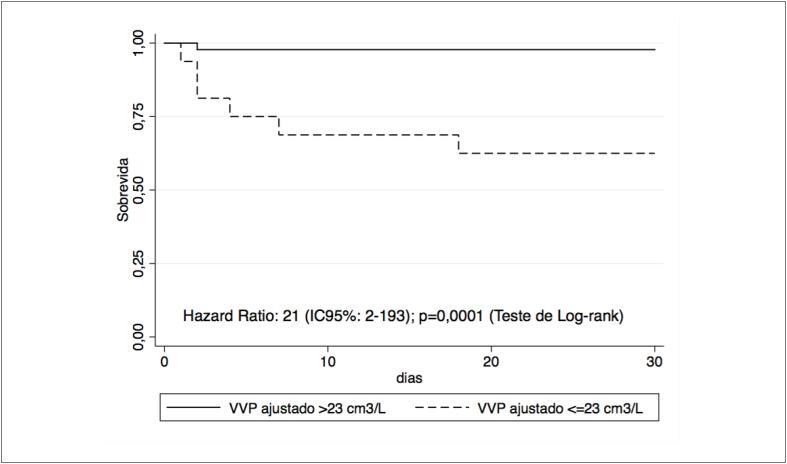
Curvas de Kaplan-Meier comparando a sobrevida em 1 mês entre os pacientes com volume vascular pulmonar (VPV) ajustado menor e maior que 23 cm3/L quantificado automaticamente por meio do software Yacta.

Na análise de sobrevida, o VVP ajustado <23 cm^3^/L foi significativamente associado a uma maior taxa de mortalidade (*hazard ratio* [HR]: 21 [IC95%: 2-193], p=0,0001) durante o seguimento de um mês (Figura 3).

**Figura 4:**
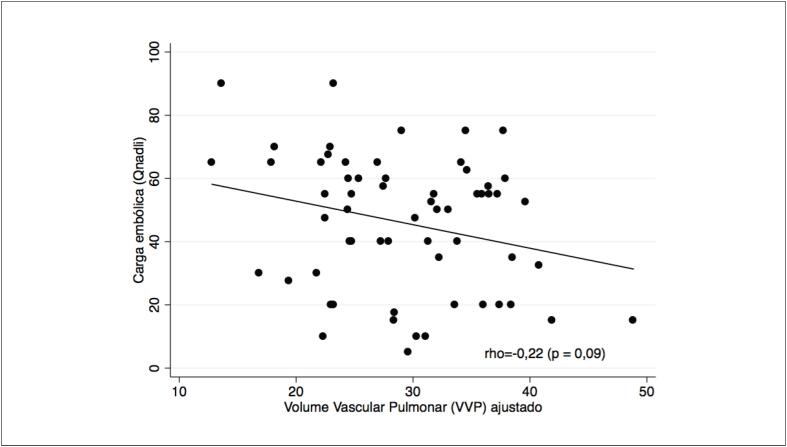
Gráfico de dispersão mostrando a correlação entre o volume vascular pulmonar (VVP) ajustado quantificado pelo software Yacta e a carga embólica quantificada manualmente de acordo com Qanadli.

A carga embólica quantificada manualmente de acordo com a descrição de Qanadli e o VVP ajustado quantificado automaticamente pelo *software* Yacta não apresentaram uma correlação significativa (Rho = –0,22, p=0,09) (Figura 4).

**Figura 5:**
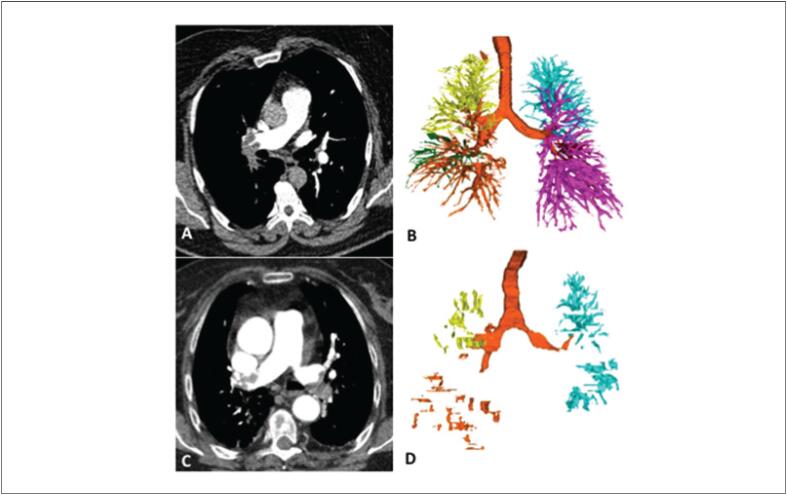
Exemplos da quantificação vascular pulmonar com o software Yacta totalmente automatizado em dois pacientes diferentes com embolia pulmonar aguda (EPA). O primeiro paciente (sobrevivente), homem, 47 anos de idade, foi diagnosticado com EPA no pulmão direito (imagem angio-CT em A) e, após segmentação e análise vascular (B), apresentou um volume vascular pulmonar (VVP) de 157 cm^3^ e um VVP ajustado de 33,7 cm^3^/L. O segundo paciente (não sobrevivente), mulher, 75 anos de idade, EPA bilateral (imagem angio-CT em C) e após segmentação e análise vascular (D) apresentou um VVP de 19 cm^3^ e VVP ajustado de 12,8 cm^3^/L.

Na Figura 5, descrevemos os exemplos da angio-CT e da quantificação dos vasos pulmonares (*software* Yacta) em dois pacientes com diferentes desfechos clínicos incluídos nesta investigação.

## Discussão

Atualmente, a angio-CT é a ferramenta mais utilizada para o diagnóstico de EPA no departamento de emergência.^5^^,^^11^ O desenvolvimento de parâmetros utilizando este método de imagem para estratificar o risco de complicações nesses pacientes é desejável e pode ajudar a individualizar o tratamento de acordo com a gravidade de cada apresentação.^4^^,^^12^ Nossa investigação mostrou que a quantificação totalmente automática do VVP ajustado em pacientes com EPA foi um preditor independente de mortalidade em 1 mês. O desempenho prognóstico dessa nova ferramenta foi superior aos parâmetros prognósticos clássicos avaliados nesse cenário por meio da angio-CT, como a razão do diâmetro do VD/VE e a carga embólica.

A elevada taxa de angio-CT positiva para EPA (53%) nesta investigação pode ser explicada pelo fato de a seleção dos pacientes ter sido realizada por meio do código do CID durante a alta hospitalar, e, provavelmente, a maioria dos pacientes cujo diagnóstico de EPA foi excluído pela angio-CT negativa não recebeu a codificação desse diagnóstico na alta hospitalar.

A razão do diâmetro do VD/VE é um parâmetro que avalia indiretamente a dilatação e a disfunção do VD observada durante a EPA.^13^ Entre os parâmetros obtidos pela angio-CT, a razão do diâmetro do VD/VE é a mais avaliada na literatura científica; apesar disso, falta padronização nos aspectos técnicos de sua mensuração e discordâncias no ponto de corte mais apropriado. A maioria dos estudos utilizou uma razão de diâmetro do VD/VE ≥1 como anormal.

Estudos isolados não mostraram a utilidade da razão do diâmetro do VD/VE ≥1 na estratificação prognóstica após a EPA. Coutance et al^.,^^6^ analisaram a angio-CT de 383 pacientes com esse diagnóstico e mostraram que a razão do diâmetro do VD/VE ≥1 não estava associada à mortalidade (OR: 1,54 [IC95%: 0,70-3,40]) e mostrou baixa sensibilidade (46% [IC95%: 27-66]), baixa especificidade (59% [IC 95%: 54-64%]) e baixo valor preditivo positivo (8% [IC95%: 5-14]) deste achado em predizer a mortalidade em 1 mês.^6^

Moroni et al.,^14^ ao analisar 225 angio-CT de pacientes com EPA não grave, a razão de diâmetro do VD/VE >1 foi apenas um preditor de mortalidade quando associada à baixa carga embólica (<40%); no entanto, na análise multivariada, a razão do diâmetro do VD/VE >1 e a forma do septo interventricular não se associou à mortalidade.^14^

Kumamaru et al.,^15^ retrospectivamente, analisaram 1.698 angio-CT de pacientes com EPA. Os parâmetros tradicionalmente avaliados também não foram associados à mortalidade por todas as causas em 1 mês. Os parâmetros avaliados foram: localização do êmbolo mais proximal (p=0,14), infarto do parênquima pulmonar (p=0,90), diâmetro do VD > VE (p=0,69), refluxo de contraste para a veia hepática (p=0,40), curvatura da septo interventricular (p=0,40), diâmetro AP/aorta (p=0,93). Por outro lado, achados não tradicionais foram preditores de mortalidade, como, por exemplo, a presença de derrame pleural e pericárdico; lesão pulmonar, hepática e óssea sugerindo malignidade, ascite etc.^15^ Provavelmente, esses achados estão muito mais relacionados ao prognóstico das doenças associadas, como o câncer, do que à própria EPA. Investigação de van der Meer et al. também não mostrou associação entre a razão do diâmetro AP/Aorta (p=0,66) e do desvio do septo interventricular (p=0,20) com a mortalidade.^16^

Somente em uma recente metanálise envolvendo um grande número de pacientes foi possível mostrar a associação prognóstica da relação VD/VE após EPA. Comparando 2.612 pacientes com uma relação anormal do diâmetro do VD/VE com 2.049 pacientes com esse parâmetro dentro da faixa de normalidade, o aumento da relação VD/VE foi associado à mortalidade em 1 mês na análise que incluiu todos os pacientes (OR: 2,08 [IC95%: 1,63-2,66] p <0,00001), e naquela em que somente pacientes com estabilidade hemodinâmica foram incluídos (OR: 1,64 [IC95%: 1,06-2,52] p=0,03).^17^ Em nossa investigação, o VVP ajustado <23 cm^3^/L apresentou melhor acurácia prognóstica do que a razão dos diâmetros VD/VE.

Os escores de obstrução da artéria pulmonar ou carga embólica obtidos por meio da angio-CT foram descritos inicialmente por Qanadli et al.,^7^ em 2001. Nesse estudo inicial, eles compararam os achados da angio-CT com a angiografia pulmonar convencional e mostraram boa concordância entre os métodos (r=0,867, p <0,0001) para quantificação do grau de obstrução. Uma carga embólica ≥40% identificou mais de 90% dos pacientes com dilatação do VD.^7^

Em estudos iniciais, como de Wu et al.^18^ e van der Meer et al.^16^ a quantificação da carga embólica na artéria pulmonar esteve associada à mortalidade.^18^^,^^16^ No entanto, estudos subsequentes falharam em mostrar uma associação desses escores de obstrução da artéria pulmonar com importantes desfechos clínicos, como a mortalidade. Kong et al.^19^ analisaram esses escores de obstrução juntamente com a presença de defeitos de perfusão pulmonar na angio-CT de 55 pacientes estratificados por meio de testes clínicos e laboratoriais em alto, intermediário e baixo risco. Os escores de obstrução não conseguiram distinguir adequadamente esses três grupos, e a quantificação dos defeitos de perfusão apresentou melhor desempenho para fazer essa discriminação.^19^ Atasoy et al.^20^ ao analisar a angio-CT de 67 pacientes, a carga embólica ≥40% não foi associada à mortalidade (OR: 0,989 [IC95%: 0,95-1,03] p=0,486).^20^ Araoz et al.^21^ avaliaram 1.193 angio-CT positivas para EPA, nem a carga embólica nem a razão do diâmetro VD/VE foram associadas à mortalidade, apenas o desvio do septo interventricular foi associado à mortalidade (OR: 1,97, p=0,05), mas com sensibilidade muito baixa (18% a 21%).^21^

Mesmo em pacientes com EPA grave admitidos na unidade de terapia intensiva, a carga embólica na artéria pulmonar, utilizando quatro sistemas de pontuação diferentes, não esteve associada à taxa de mortalidade durante a internação hospitalar.^22^

Em nossa investigação, a carga embólica também não foi um preditor de mortalidade em 1 mês. Embora sejam variáveis inter-relacionadas, o VVP ajustado foi um preditor independente de mortalidade em 1 mês nesses pacientes com EPA. Esse fato pode ser explicado pelos problemas técnicos na quantificação manual da carga embólica, restrita principalmente à avaliação dos vasos de maior calibre. O *software* Yacta permite uma melhor avaliação da obstrução dos vasos de pequeno calibre e poderia refletir mais adequadamente o prognóstico após a EPA.

Algumas limitações do nosso estudo merecem ser consideradas. Primeiro, o *software* Yacta não conseguiu medir adequadamente os volumes vasculares pulmonares em 27% dos pacientes, principalmente devido à presença de artefatos. No entanto, aprimoramentos do *software* e da aquisição de imagens podem reduzir essa falha. A aquisição da imagem da angio-CT sincronizada pelo eletrocardiograma pode melhorar a qualidade da imagem e permitir um melhor desempenho desse *software*. Segundo, essa investigação teve um tamanho amostral pequeno que talvez tenha sido insuficiente para avaliar o efeito preditivo dos parâmetros clássicos da angio-CT, como da razão do diâmetro VD/VE. Entretanto, mesmo com esse tamanho amostral reduzido, o VVP ajustado foi um forte preditor de mortalidade, levando à possível extrapolação de que esse parâmetro, de fato, apresente melhor acurácia prognóstica. Terceiro, houve uma tendência estatística na correlação entre a carga embólica quantificada manualmente e o VVP ajustado; o tamanho reduzido da amostra poderia explicar essa falta de correlação estatisticamente significativa. Quarto, este novo parâmetro precisa ser avaliado em outros estudos multicêntricos e prospectivos. Em quinto lugar, nesta investigação, apenas os parâmetros da angio-CT foram analisados, a inclusão de outros parâmetros como escore PESI, troponina, NT-proBNP, junto com os achados da imagem dentro de algoritmos de estratificação de risco de EPA precisa ser futuramente investigado.^23^^,^^24^ Sexto, a detecção de vasos pelo programa é baseada não apenas em valores de atenuação, mas também na análise tridimensional da anatomia vascular; a presença de opacidades pulmonares não impede a análise correta do VVP. O algoritmo de segmentação da Yacta é muito robusto e eficaz, porque utiliza ferramentas diferentes para identificar os pulmões, as vias aéreas e os vasos. O que pode alterar a vasculatura pulmonar é a presença de doença das vias aéreas e enfisema, que pode levar à vasoconstrição hipóxica ou destruição vascular, confundindo com trombose/embolia. Apesar disso, nossa investigação teve uma baixa prevalência de pacientes com diagnóstico de doença pulmonar obstrutiva crônica. Finalmente, a mortalidade por todas as causas foi o desfecho avaliado; contudo, não necessariamente secundária à EPA, mas, na maioria das investigações que avaliaram os parâmetros da angio-CT, apenas a mortalidade por todas as causas foi avaliada.

## Conclusão

O VVP ajustado estimado pelo *software* Yacta parece ser uma ferramenta promissora para a estratificação prognóstica após EPA, principalmente quando comparado a outros parâmetros prognósticos clássicos da angio-CT.
