# Supplementary material for: Volume Vascular Pulmonar Estimado por Software Automatizado é um Preditor de Mortalidade após Embolia Pulmonar Aguda
Source: Arq Bras Cardiol. 2020 Nov 1;115(5):809–18. [Article in Portuguese] doi: 10.36660/abc.20190392 (PMC8452195; doi:10.36660/abc.20190392)
Supplement: Translation

# Translation: Pulmonary Vascular Volume Estimated by Automated Software is a Mortality Predictor after Acute Pulmonary Embolism

**Authors:** Leonardo Soriano, Marcel Koenigkam Santos, Danilo Tadeu Wada, Kelvin Vilalva, Talita Tavares Castro, Oliver Weinheimer, Valdair Francisco Muglia, Antonio Pazin, Carlos Henrique Miranda

**Affiliations:** 1 Ribeirão PretoSP Brazil Universidade de São Paulo Faculdade de Medicina de Ribeirão Preto - Divisão de Medicina de Emergência do Departamento de Clínica Médica, Ribeirão Preto, SP - Brazil; 2 Ribeirão PretoSP Brazil Universidade de São Paulo Faculdade de Medicina de Ribeirão Preto - Divisão de Radiologia do Departamento de Imagens Médicas, Hematologia e Oncologia Clínica, Ribeirão Preto, SP - Brazil; 3 Heidelberg Germany University Hospital Heidelberg - Department of Diagnostic and Interventional Radiology and Translational Lung Research Centre Heidelberg (TLRC) - German Lung Research Centre (DZL), Heidelberg – Germany

**Keywords:** Pulmonary Embolism, Tomography Computed, Prognosis, Diagnostic Imaging, Pulmonary Circulation, Emergency Medical Services, Mortality

## Abstract

**Background:**

Acute pulmonary embolism (APE) has a variable clinical outcome. Computed tomography pulmonary angiography (CTPA) is the gold standard for this diagnosis.

**Objective:**

To evaluate if the pulmonary vascular volume (PVV) quantified by automated software is a mortality predictor after APE.

**Methods:**

Retrospective cohort study where the CTPA imaging of 61 patients with APE was reanalyzed. Pulmonary vascular volume (PVV) and pulmonary volume (PV) were automatically estimated using the Yacta software. We calculated the adjusted PVV by the ratio: PVV(cm^3^)/PV(liters). Classical prognostic CTPA parameters (clot load index, right ventricle/left ventricle diameter ratio, pulmonary artery/aorta diameter ratio, ventricular septal bowing, pulmonary infarction and reflux of contrast into the hepatic vein) were assessed. The outcome assessed was one-month mortality. We considered a p-value <0.05 as statistically significant.

**Results:**

Seven deaths (11%) occurred at one month among these 61 patients. PVV<23cm^3^/L was an independent predictor of one-month mortality in the univariate [odds ratio (OR): 26; 95% confidence interval (CI): 3-244; p=0.004] and multivariate analyses [adjusted OR: 19; 95%CI: 1.3-270; p=0.03]. The classical CTPA parameters were not associated with one-month mortality in this sample. The PVV<23cm^3^/L showed a sensitivity of 86%, a specificity of 82%, a negative predictive value of 94% and a positive predictive value of 64% to identify the patients who died.

**Conclusion:**

PVV<23cm^3^/L was an independent predictor of one-month mortality after APE. This parameter showed better prognostic performance than other classical CTPA findings. (Arq Bras Cardiol. 2020; 115(5):809-818)

## Introduction

Acute pulmonary embolism (APE) is a significant cause of dyspnea and chest pain in the emergency department.^1^ The prognosis after an event is extremely variable. The majority of patients have an excellent clinical course. However, some patients may have a catastrophic clinical course developing into circulatory shock, cardiac arrest, and death.^2^ Due to this heterogeneous clinical presentation, some parameters are used for prognostic stratification to allow more intensive surveillance among patients with a higher probability of complications. Currently, computed tomographic pulmonary angiography (CTPA) is the gold standard among diagnostic methods. Because of this, CTPA parameters are assessed to help in the prognostic stratification and the decision-making regarding the treatment.^3^^–^^5^

The most frequent CTPA parameter used for prognostic stratification is the right ventricle enlargement, which is mainly identified through the right ventricle/left ventricle (LV) diameter ratio≥1.^6^ The clot load index, manually quantified as described by Qanadli, when higher than 40% aids to identify patients with right ventricular dilation.^7^ However, in clinical practice, these isolated parameters have a weak association with mortality and shock development. Because of this, the guidelines recommend that these parameters should not be used alone and only combined with other prognostic markers, such as troponin and N-terminal pro–B type natriuretic peptide (NT-proBNP).^8^

The objective of this investigation was to assess if fully automatic pulmonary vascular volume quantification using CTPA is a mortality predictor after APE and to compare its prognostic performance with other classical CTPA parameters in predicting the one-month mortality.

## Methods

Single-center, retrospective cohort study that included patients with a primary diagnosis of APE admitted to our emergency department. Our hospital is exclusively dedicated to high-complexity emergency, and it has around 3000 medical appointments per month. This study was approved by the Research Ethics Committee of our institution and followed the Declaration of Helsinki.

### Patients

Medical records of adult patients (>18 years old) admitted from January 2009 to December 2015 were reviewed. These patients had a primary diagnosis of APE, registered at hospital discharge through the codes I26.0 (pulmonary embolism with acute cor pulmonale) and I26.9 (pulmonary embolism without acute cor pulmonale), according to the International Statistical Classification of Diseases (ICD-10). The definitive diagnosis of APE was defined as the presence of compatible clinical condition associated with at least one criteria, which could be: CTPA with filling defects; or pulmonary ventilation and perfusion scintigraphy with perfusion defects in ventilated areas (high probability); or conventional pulmonary angiography with intraluminal filling defect; or lower-limb ultrasonography compatible with deep vein thrombosis; or necropsy with high thrombotic load in the pulmonary artery without evidence for other alternative diagnoses.

Demographic and clinical data were obtained by reviewing medical records. We used the diagnosis reported by the patient and included in the medical record. The outcome evaluated in this investigation was one-month all-cause mortality. For those patients who were discharged before completing 30 days, a nurse from the clinical research unit of our institution, who was trained to evaluate survival, made a telephone call, and when the occurrence of death was verified, the date of the event was requested.

### CTPA technique and interpretation

CTPA was performed using multidetector CT (MDCT) scanners, and volumetric images were obtained after intravenous administration of iodinated contrast using a single bolus injection followed by a flush of saline solution and a bolus detection technique to identify pulmonary artery enhancement. Other typical parameters used were: slice thickness ≤ 2.5 mm, reconstruction interval of 1 mm, kVp of 120, mAs reference of 150-220, gantry rotation of 0.3 to 0.7s. Volumetric acquisitions were reconstructed with soft and hard filters. Two chest radiologists reanalyzed the images after retrieving them using the DICOM (Digital Imaging and Communication in Medicine) format, in calibrated and dedicated workstations. Both radiologists were blind to the clinical evolution of these patients.

We analyzed the classical prognostic parameters of CTPA described in the medical literature. RV/LV axial diameter ratio was obtained by measuring the short axes of the ventricles in the axial plane at their posterior third. An RV/LV diameter cutoff ratio of 1 was used as recommended in the literature. The transverse diameter of the main pulmonary artery (PA) and the transverse diameter of the ascending aorta at the same level were measured. Ventricular septal bowing was considered if there was both septal flattening and septum deviation convex toward the left ventricle. The presence of contrast reflux into the hepatic veins was also assessed. The presence of pulmonary infarction was defined if a pleural-based parenchymal opacity with convex, bulging borders and linear strands directed from the apex towards the hilum was identified. The clot load index was calculated using the method described by Qanadli et al.^7^ An index higher than 60% was considered to indicate a high embolic burden.

The quantitative vascular analysis of CTPA imaging was carried out with the academic program Yacta (Heidelberg University, Heidelberg, Germany), version 2.7. The Yacta software works entirely automatically, requiring no user intervention at any stage of the process. Imaging analysis lasts about 10 minutes. Initially, Yacta segments (anatomically separate) the airways, blood vessels, lungs and, their lobes, then supply lung volumes and densities, together with the volume of blood vessels. This software uses an attenuation coefficient of −500 HU as the standard threshold for detection of vessels. In lungs with modified attenuation coefficients, Yacta calculates a new threshold based on the histogram. Intrapulmonary voxels with coefficients above the calculated threshold are then marked as vessels, and vessels with three-dimensional communication larger than 100 mm^3^ are considered in the analysis.^9^^,^^10^ Yacta software estimated the pulmonary volume (PV) in liters (L) and the pulmonary vascular volume (PVV) in cm^3^. Since the PVV has a variation according to lung size, we performed an adjustment through the ratio: PVV (cm^3^) /PV (L).

### Statistical analysis

We used the Shapiro-Wilk test to evaluate the type of variable distribution. Categorical variables were expressed as percentages. Continuous variables with normal distribution were expressed as mean and standard deviation, and the other variables were expressed as median and interquartile range (25^th^ percentile, 75^th^ percentile). The chi-square test was used to compare two categorical variables. The unpaired Student's T-test was used to compare two continuous variables with normal distribution and the Mann-Whitney test to compare two continuous variables with non-normal distribution. In the univariate analysis, the odds ratio (OR) and its respective 95% confidence interval (95%CI) were calculated for each parameter, followed by the chi-square test. For the multivariate analysis, a logistic regression model was used, with adjustment for the variables: age, pulmonary embolism severity index (PESI), respiratory rate, cardiac arrest, and circulatory shock. Spearman's rank test was used to evaluate the correlation between two continuous variables. The area under the receiver operating characteristic (ROC) curve was used to compare the prognostic accuracy of each CTPA parameter. We used the Youden index to determine the best cutoff point of the adjusted PVV to identify the patients who died. The cutoff point standardized in the medical literature was used for other CTPA parameters. In the survival analysis, the Kaplan-Meier curves were compared through the log-rank test. A p-value <0.05 was considered as statistically significant. The software STATA 13.1 (College Station, TX, USA) was used for the statistical analysis.

## Results

Of the 231 individuals with suspected APE assessed in the emergency department, the diagnosis was confirmed in 123 patients (53%). The diagnosis was attained through the CTPA in 99 patients (80%). Considering the patients who underwent CTPA, the imaging was retrieved for reanalysis in 84 of them. Automated pulmonary vascular volume determination using the Yacta software was possible in 61 of these recovered image files. Flow charts of the patients included in this investigation and the reasons that made the Yacta analysis impossible are shown in Figure 1.

**Figure 1:** Flow chart showing the criteria selection for the patients included in this investigation.

The baseline characteristics of these 61 patients are shown in table 1. Of these patients, 07 (11%) died in one month. When comparing non-survivors (n=7) with survivors (n=54), there were no significant differences between these two groups, except a higher respiratory rate in the non-survivors' group (31±7 cycles/min vs. 33±7 cycles/min, p=0.01).

**Table 1:** Baseline characteristics of the patients divided according to the one-month mortality

Parameter	Survivors n=54	Non-Survivors n=7	p
**Demographic data**			
Age, years (mean±sd)	54±16	61±17	0.34
Age>65years-old, n.(%)	19(35)	3(43)	0.69
Gender male, n.(%)	24(44)	2(29)	0.42
Clinical presentation			
Cardiac arrest, n.(%)	2(04)	1(14)	0.22
Circulatory shock, n.(%)	5(09)	2(28)	0.13
Dyspnea, n.(%)	46(85)	7(100)	0.27
Hemoptysis, n.(%)	7(13)	0(00)	0.31
Syncope, n.(%)	13(24)	0(00)	0.14
Cough, n.(%)	20(37)	3(43)	0.76
Pleuritic chest pain, n.(%)	17(31)	4(57)	0.18
Fever, n.(%)	7(13)	2(28)	0.27
Wells score, (median, 25^th^−75^th^)	4.5 (3.0-7.0)	4.0 (1.5-4.5)	0.17
PESI score, (median, 25^th^−75^th^)	78 (65-108)	97 (95-108)	0.13
Symptom duration, days (median, 25^th^−75^th^)	3(1-6)	2(1-6)	0.29
Predisposing factors			
Previous PE/DVT, n.(%)	11(20)	0(00)	0.19
Active cancer, n.(%)	4(07)	2(28)	0.07
Recent surgery, n.(%)	7(13)	0(00)	0.31
Immobilization, n.(%)	13(24)	1(14)	0.56
Fracture, n.(%)	7(13)	0(00)	0.31
Previous stroke, n.(%)	7(13)	1(14)	0.92
Contraceptive use, n.(%)	7(13)	0(00)	0.31
Obesity, n.(%)	23(43)	3(43)	0.91
Heart failure, n.(%)	7(13)	0(00)	0.31
COPD, n.(%)	4(07)	1(14)	0.53
Thrombophilia, n.(%)	5(09)	1(14)	0.67
Physical examination			
Heart rate; bpm, (mean±sd)	94±16	106±23	0.07
Respiratory rate, cycles/min (mean±sd)	23±7	31±7	0.01
Respiratory rate > 20 cycles/min, n.(%)	36(67)	6(86)	0.12
SBP, mmHg (mean±sd)	123±28	113±14	0.37
DBP, mmHg (mean±sd)	75±14	69±19	0.32
Laboratory tests			
Creatinine, mg/dL (mean±sd)	1.08±0.27	1.16±0.83	0.59
Hemoglobin, g/dL (mean±sd)	13±2	12±3	0.05
Oxygen Saturation, % (mean±sd)	92±7	87±8	0.09
Troponin I, μg/L (mean±sd)	0.16±0.29	0.13±0.12	0.79
NT-proBNP, μg/L(mean±sd)	2604±3040	3433±2343	0.60
Treatment			
Thrombolytic, n.(%)	14(26)	2(29)	0.88
Unfractionated heparin, n.(%)	7(13)	1(14)	0.81
Low molecular weight heparin, n.(%)	38(70)	6(86)	0.12

PESI: pulmonary embolism severity index; PE: pulmonary embolism; DVT: deep vein thrombosis, COPD: chronic obstructive pulmonary disease; SBP: systolic blood pressure; DBP: diastolic blood pressure, NT-proBNP: N-terminal type B natriuretic peptide.

Regarding the CTPA parameters analysis, the pulmonary vascular volume (PVV) and the adjusted PVV were significantly decreased in the non-survivors group in comparison to the survivor's group (56±24 cm^3^ vs. 88±32 cm^3^, p=0.015 and 21±6 cm^3^/L vs. 30±7 cm^3^/L, p=0.001, respectively). The other parameters evaluated by the CTPA (clot load index, RV/LV axial diameter ratio, PA/Aorta diameter ratio, ventricular septal bowing, pulmonary infarction, and contrast reflux into the hepatic vein) did not differ significantly between these two groups (Table 2).

**Table 2:** Computed tomography pulmonary angiography (CTPA) findings divided according to the one-month survival rate

Parameter	Survivors n=54	Non-survivors n=7	p
Yacta parameters			
Pulmonary volume (L), mean±sd	2.91±0.90	2.73±1.31	0.64
Pulmonary vascular volume (cm^3^), mean±sd	88±32	56±24	0.01
Adjusted pulmonary vascular volume (cm^3^/L), mean±sd	30±7	21±6	0.001
Classical CTPA parameters			
Clot load index (%), mean±sd	47±21	40±26	0.40
Central clot, n. (%)	5 (09)	2(28)	0.13
Bilateral clot, n. (%)	45(83)	5(72)	0.59
Unilateral clot, n. (%)	4(08)	0(00)	1.00
RV/LV axial diameter ratio, mean±sd	1.20±0.36	1.25±0.28	0.74
RV/LV axial diameter ratio>1, n.(%)	36(67)	6(86)	0.30
PA/Aorta diameter ratio, mean±sd	0.91±0.17	0.91±0.90	0.92
Ventricular septal bowing (VSB), n. (%)	32(59)	5(71)	0.53
Pulmonary infarction, n. (%)	25(46)	2(29)	0.37
Reflux of contrast into the hepatic vein, n. (%)	20(37)	3(43)	0.76

RV: right ventricle; LV: left ventricle; PA: pulmonary artery

The analysis using the area under the ROC curve (AUC), the 1/adjusted PVV showed the best prognostic accuracy performance with an AUC of 0.86 (95%CI: 0.68-1.00) compared to the other continuous variables [RV/LV diameter ratio with AUC of 0.56 (95%CI: 0.37-0.75), the PA/Aorta diameter with AUC of 0.55 (95%CI: 0.35-0.75) and the clot load index with AUC of 0.44 (95%CI: 0.16-0.74)], p<0.01(Figure 2).

**Figure 2:** ROC-curves showing the prognostic performance of the continuous CTPA parameters (clot load index, RV/LV diameter ratio, PA/Aorta diameter ratio) compared to the adjusted PVV in predicting one-month mortality after APE. CTPA: computed tomographic pulmonary angiography; RV: right ventricle; LV: left ventricle; PA: pulmonary artery; PVV: pulmonary vascular volume; APE: acute pulmonary embolism.

The best cutoff point of the adjusted PVV to determine the one-month mortality was 23 cm^3^/L [sensitivity: 86%(95%CI: 42-99), specificity: 82%(95%CI: 69-91), positive predictive value: 64%(95%CI: 49-77) and negative predictive value: 94%(95%CI: 70-99)].

In the univariate analysis, the adjusted PVV<23 cm^3^/L [odds ratio (OR): 26 (95%CI: 3-244), p=0.004] and the respiratory rate [OR: 1.1(95%CI: 1.01-1.26), p=0.03] were the one-month mortality predictors. In the multivariate analysis, only the PVV<23 cm^3^/L remained as an independent predictor of one-month mortality [adjusted OR: 19 (95%CI: 1.3-270), p=0.03]. The classical prognostic CTPA parameters were not associated with one-month mortality (Table 3).

**Table 3:** Predictors of one-month mortality after APE in the univariate and multivariate analysis

Parameters	Univariate				Multivariate		
	OR	95%CI	p		OR	95%CI	p
Demographic/ clinical data							
Age	1.0	0.97- 1.0	0.34				
Gender	0.5	0.09-2.8	0.43				
Active cancer	5.0	0.73-34.5	0.10				
Circulatory shock	3.9	0.60-25.7	0.15				
Cardiac arrest	4.3	0.34-55.2	0.26				
Heart rate	1.0	0.99-1.1	0.09				
Respiratory rate	1.1	1.01-1.26	0.03		1.56	0.95-2.57	0.08
PESI score	1.0	0.99-1.00	0.12				
Imaging							
Adjusted pulmonary vascular volume ≤ 23 cm^3^/L	26.0	3.0-244	0.004		19.0	1.3-279.0	0.03
Clot load index	0.9	0.95-1.0	0.44				
Clot load index ≥ 40%	0.5	0.0-2.3	0.36				
Clot load index ≥ 60%	2.6	0.5-13.4	0.24				
RV/LV diameter ratio	1.5	0.2-12.6	0.73				
RV/LV diameter ratio ≥1	3.0	0.3-26.8	0.32				
Ventricular septal bowing	1.7	0.3-9.6	0.53				
Pulmonary infarction	0.5	0.8-2.6	0.38				
Reflux of contrast into the hepatic vein	1.3	0.2-6.3	0.76				

OR: odds ratio; CI: confidence interval; PESI: pulmonary embolism severity index; RV: right ventricle; LV: left ventricle.

In the survival analysis, the PVV<23 cm^3^/L was significantly associated with a higher mortality ratio [hazard ratio (HR): 21 (95%CI:2-193), p=0.0001] during the one-month follow-up(Figure 3).

**Figure 3:** Kaplan-Meier curves comparing the one-month survival between the patients with adjusted pulmonary vascular volume (PVV) lower and higher than 23 cm^3^/L.

The clot load index manually quantified according to the Qanadli description and the adjusted PVV quantified automatically through the Yacta software did not show a significant correlation [Rho=−0.22, p=0.09] (Figure 4).

**Figure 4:** Scatter plot showing the association between the adjusted pulmonary vascular volume (PVV) quantified through the Yacta software and the clot load index manually quantified according to Qanadli.

Figure 5 depicts the CTPA and the pulmonary vessel quantification imaging (Yacta software) examples in two patients with different clinical outcomes included in this investigation.

**Figure 5:** Examples of fully automated pulmonary vascular quantification using the Yacta software in two different patients with acute pulmonary embolism (APE). The first patient (survivor), man, 47 years old, was diagnosed with APE in the right lung (CTPA image in A) and after vascular segmentation and analysis (B) showed a pulmonary vascular volume (PVV) of 157 cm^3^ and an adjusted PVV of 33.7 cm^3^/L. The second patient (non-survivor), woman, 75 years old, had a bilateral APE (CTPA image in D) and after lung (E) and vascular segmentation (F) showed a pulmonary vascular volume (PVV) of 19 cm^3^ and an adjusted PVV of 12.8 cm^3^/L.

## Discussion

Currently, CTPA is the most often used tool for APE diagnosis in the emergency department.^5^^,^^11^ The development of parameters using CTPA to stratify the risk of complications in these patients is desirable and could help to individualize the treatment according to the severity of each presentation.^4^^,^^12^ Our investigation showed that a fully automatic quantification of adjusted PVV in patients with APE was an independent predictor of one-month mortality. The prognostic performance of this new tool was superior to the classical prognostic CTPA parameters evaluated in this setting, such as the RV/LV diameter ratio and clot load index.

The high rate of positive CTPA for APE (53%) in this investigation can be explained because the selection of patients was performed through the ICD code during the hospital discharge and, probably, in the majority of patients in whom the PE diagnosis was excluded through negative CTPA; the ICD of acute pulmonary embolism was not included during the discharge, and these patients were not identified.

The RV/LV diameter ratio is a parameter that indirectly evaluates right ventricular dilation and RV dysfunction observed during the APE.^13^ Among the parameters obtained by the CTPA, the RV/LV diameter ratio is the most frequently evaluated in the scientific literature; despite this fact, there is lack of standardization regarding the technical aspects of its measurement and disagreements about the most appropriate cutoff point. Most of the studies used an RV/LV diameter ratio ≥ 1 as abnormal.

Isolated studies have not demonstrated the usefulness of RV/LV diameter ratio ≥1 in the prognostic stratification after APE. Coutance et al.^6^ analyzing the CTPA of 383 patients with this diagnosis, showed that the RV/LV diameter ratio ≥1 was not associated with mortality [OR: 1.54; 95%CI: 0.70-3.40], had a low sensitivity [46%; 95%CI: 27-66], a low specificity [59%; 95%CI: 54-64%] and low positive predictive value [08%; 95%CI: 5.0-14.0] in predicting the one-month mortality.^6^

Moroni et al.^14^ when analyzing 225 CTPA of patients with non-severe APE, observed that the RV/ LV diameter ratio > 1 was only a predictor of mortality when associated with low embolic burden (<40%), but in the multivariate analysis, the RV/LV diameter ratio > 1 and the shape of interventricular septum were not associated with death.^14^

Kumamaru et al.^15^ retrospectively analyzed 1698 CTPAs in patients with APE. The traditionally evaluated parameters were also not associated with all-cause mortality at one month. The parameters assessed were: the location of the most proximal embolus (p=0.14), parenchymal infarction (p=0.90), RV> LV diameter (p= 0.69), contrast reflux to the hepatic vein (p=0.40), bowing of the septum (p=0.40), and PA/Aorta diameter (p=0.93). On the other hand, nontraditional findings were predictors of mortality, such as pleural and pericardial effusion; lung, liver and bone lesion suggesting malignancy, ascites, etc.^15^ These findings are probably much more related to the prognosis of associated diseases such as cancer than the APE itself. An investigation by van der Meer *et al.* also showed no association between the PA/Aorta diameter ratio (p=0.66) and the presence of ventricular septal bowing (p=0.20) with mortality.^16^

A recent meta-analysis involving a large number of patients was able to demonstrate the prognostic association of the RV/LV ratio after APE. When comparing 2612 patients with abnormal RV/LV diameter ratio with 2049 patients who had this parameter within the regular range, the increased RV/LV ratio showed to be associated with the one-month mortality in the analysis that included all patients [OR: 2.08; 95%CI: 1.63-2.66; p <0.00001], and which included only patients with hemodynamic stability [OR: 1.64; 95%CI: 1.06-2.52; p=0.03].^17^ In our investigation, the adjusted PVV<23 cm^3^/L showed a better prognostic performance than the RV/LV diameter ratio.

The pulmonary artery obstruction scores or clot load index obtained through CTPA were initially described by Qanadli et al.^7^ in 2001. In this initial study, they compared CTPA findings with invasive pulmonary angiography and showed good agreement between the methods (r = 0.867, p <0.0001) for the quantification of the obstruction degree. A clot load index ≥ 40% identified more than 90% of patients with RV dilation.^7^

In early studies, such as the ones by Wu et al.^18^ and van der Meer et al.^16^ the quantification of the pulmonary artery embolic obstruction was associated with mortality.^18^^,^^16^ However, subsequent studies failed to demonstrate an association of these pulmonary artery obstruction scores with important clinical outcomes, such as mortality. Kong et al.^19^ analyzed these obstruction scores together with the presence of pulmonary perfusion defects in the CTPA of 55 patients stratified through clinical and laboratory tests as high, intermediate, and low-risk. The obstruction scores failed to differentiate these three groups adequately, and the quantification of perfusion defects showed a better performance to make this discrimination.^19^ Atasoy et al.^20^ when analyzing the CTPA of 67 patients, observed that a clot load index ≥ 40% was not associated with mortality [OR: 0.989; 95%CI: 0.95-1.03; p = 0.486].^20^ Araoz et al.^21^ evaluated 1193 CTPAs positive for APE, and observed that neither the thrombotic burden nor the RV/LV diameter ratio was associated with mortality, and only ventricular septal bowing was associated with mortality [OR: 1.97, p=0.05], albeit with very low sensitivity (18-21%).^21^

Even in patients with severe APE admitted to the intensive care unit, the clot load in the pulmonary artery using four different scoring systems was not associated with the mortality rate during the hospital stay.^22^

In our investigation, the clot load index was also not a predictor of one-month mortality, although they are interrelated variables; the adjusted PVV was an independent predictor of one-month mortality in these patients with APE. This fact could be explained by the technical troubles in the manual quantification of the clot load, which is mainly restricted to the evaluation of the larger-caliber vessels. The Yacta software allowed a better evaluation of the small-caliber vessel obstruction, and it could more adequately reflect the prognosis after APE.

Some limitations of our study deserve to be considered. First, the Yacta software was not able to adequately measure pulmonary vascular volumes in 27% of patients, mainly due to the presence of artifacts. However, software improvements and enhancements in imaging acquisition may reduce this failure. The use of ECG-gated CTPA can improve the imaging quality and allow better performance of this software. Second, this investigation had a small sample size, and maybe it was underpowered to evaluate the predictive effect of the classical CTPA parameters, such as the RV/LV diameter ratio. However, even in this small sample size, the adjusted PVV was a strong predictor of mortality, leading to a possible understanding that this parameter had a better prognostic performance. Third, there was a statistical tendency in the correlation between the manually quantified clot load index and the adjusted PVV; the small sample size could explain this lack of significant correlation. Fourth, this new parameter needs to be evaluated in other multicenter and prospective studies. Fifth, in this investigation, only the CTPA parameters were analyzed, and the inclusion of these imaging findings in the APE management algorithm associated with other instruments, such as the pulmonary embolism severity index (PESI) and biomarkers such as troponin or NT-proBNP need to be further evaluated.^23^^,^^24^ Sixth, vessel detection by the program is based not only on attenuation values but also on the three-dimensional analysis of vascular anatomy; the presence of pulmonary opacities does not preclude the correct analysis of the vascular volume. The Yacta segmentation algorithm is very robust and effective because it uses different tools to identify the lungs, airways, and vessels. What can alter the pulmonary vasculature is the presence of airway disease and emphysema, which can lead to hypoxic vasoconstriction or vascular destruction, and can be confounded with thrombosis/embolism. Despite this fact, our investigation had a low prevalence of patients with COPD. Finally, all-cause mortality was the evaluated outcome, although not necessarily secondary to APE; however, in the majority of the studies that evaluated these CTPA parameters, only all-cause mortality was assessed.

## Conclusion

Adjusted PVV, estimated using the Yacta software, seems to be a promising tool for the prognostic stratification after APE, mainly when compared to other classical prognostic CTPA parameters.
